# Performance of Deep Learning Models in Endoscopic Severity Assessment of Inflammatory Bowel Disease: A Systematic Review

**DOI:** 10.7759/cureus.108178

**Published:** 2026-05-03

**Authors:** Afraa Mohammed, Ahmed Mohammed, Abdelrahman Elshiekh, Sumaia Talballah, Wadah Mohamed Hussein Mohamed, Ibrahim D Mohammedallayla, Mohammed Ali, Elkhansaa A Elsamani

**Affiliations:** 1 Gastroenterology and Hepatology, Sudan National Center for Gastrointestinal and Liver Diseases, Khartoum, SDN; 2 Gastroenterology, St. Columcille's Hospital, Dublin, IRL; 3 Internal Medicine, Dr. Soliman Fakeeh Hospital, Riyadh, SAU; 4 Pathology, Najran University, Najran, SAU; 5 Internal Medicine, Sligo General Hospital, Sligo, IRL; 6 Internal Medicine, Sultan Qaboos University Hospital, Muscat, OMN; 7 Internal Medicine, Najran Armed Forces Hospital, Najran, SAU; 8 Faculty of Medicine, Al Neelain University, Khartoum, SDN; 9 Internal Medicine, Galway University Hospital, Galway, IRL

**Keywords:** artificial intelligence, crohn's disease, deep learning, endoscopy, inflammatory bowel disease, severity assessment, ulcerative colitis

## Abstract

Endoscopic severity assessment is fundamental to the management of inflammatory bowel disease (IBD). However, traditional scoring systems are limited by significant interobserver variability. Deep learning has emerged as a promising tool for objective and reproducible analysis of endoscopic images and videos. This systematic review evaluates the performance of deep learning models for endoscopic severity assessment in IBD.

We conducted a comprehensive literature search across PubMed, Scopus, Embase, and Web of Science for studies published between 2021 and 2025. We included studies that evaluated deep learning models for endoscopic severity assessment in patients with ulcerative colitis or Crohn's disease. Two reviewers independently performed data extraction and risk of bias assessment using the Quality Assessment of Diagnostic Accuracy Studies (QUADAS-2) tool. Due to heterogeneity across studies, we conducted a narrative synthesis.

Twelve studies met the inclusion criteria. Deep learning models demonstrated high diagnostic accuracy for both ulcerative colitis and Crohn's disease. Architectures ranged from convolutional neural networks to Vision Transformers. For ulcerative colitis, models achieved the area under the receiver operating characteristic curve (AUROC) values up to 0.966 for distinguishing Mayo endoscopic subscore grades. They also achieved weighted F1-scores up to 0.731 for the Ulcerative Colitis Endoscopic Index of Severity scoring. For Crohn's disease, models achieved AUROC values up to 0.971 for stricture detection and accuracy up to 98.6% for lesion classification. One study reported a 97% reduction in images requiring expert review and 87-94% faster reading times. Risk of bias assessment revealed that nine studies had a low overall risk of bias, while three had an unclear risk due to insufficient reporting.

Deep learning models consistently achieve high performance in endoscopic severity assessment of IBD. Emerging evidence supports their potential to standardize scoring, improve correlation with patient-centered outcomes, and enhance clinical workflow efficiency. Future research should prioritize external validation across diverse populations, standardized reporting, and prospective evaluation of clinical implementation and long-term outcomes.

## Introduction and background

Inflammatory bowel disease (IBD), encompassing Crohn's disease (CD) and ulcerative colitis (UC), is a chronic relapsing condition characterized by intestinal inflammation and progressive mucosal damage [1]. Accurate assessment of disease severity is essential for guiding treatment decisions, monitoring response to therapy, and predicting long-term outcomes. Endoscopy remains the gold standard for evaluating mucosal inflammation. Validated scoring systems are widely used in clinical practice and research, such as the Mayo Endoscopic Score (MES) (which assesses features like vascular pattern, bleeding, and ulceration) and the Simple Endoscopic Score for Crohn's Disease (SES-CD) [2]. However, endoscopic interpretation is inherently subjective. This often leads to significant interobserver and intraobserver variability, even among experienced gastroenterologists. Such variability may compromise clinical decision-making and highlights the need for more objective, reproducible assessment methods [3].

Recent advances in artificial intelligence (AI), particularly deep learning, have shown great promise in addressing these limitations [4]. Unlike traditional computer vision approaches, which rely on manually programmed rules and handcrafted features (e.g., edge detection or color thresholds defined by human engineers), deep learning models automatically learn to extract relevant features directly from raw image data. This end-to-end learning capability allows them to identify subtle and complex patterns that may not be easily specified by human experts. Convolutional neural networks (CNNs), a class of algorithms inspired by the human visual system, have become particularly successful for medical image analysis by detecting hierarchical features ranging from simple edges to complex anatomical structures [2]. More advanced architectures, such as Vision Transformers (ViT) (which capture global contextual relationships across an entire image using self-attention mechanisms) and spatio-temporal models (which analyze patterns across both space and time in video data), are also emerging. These models have been increasingly applied to tasks such as lesion detection, disease severity classification, and prediction of histological remission [5]. The year 2021 marked a pivotal turning point for medical AI, driven by breakthroughs such as the introduction of ViT adapted for medical imaging and the rise of self-supervised learning, techniques that allow models to learn from unlabeled data, substantially reducing reliance on expensive expert annotations. Early studies from this period demonstrated encouraging performance, with some models achieving accuracy comparable to expert endoscopists [6]. Furthermore, integrating deep learning into endoscopic workflows could enhance real-time decision-making, reduce diagnostic variability, and improve clinical efficiency.

Despite the growing body of literature, considerable heterogeneity remains across study designs, datasets, model architectures, and reported performance metrics. This makes it challenging to draw definitive conclusions about clinical utility. Concerns also persist regarding generalizability, external validation, and interpretability of deep learning models. Therefore, a comprehensive synthesis of current evidence is warranted. This systematic review aims to evaluate the performance of deep learning models for endoscopic severity assessment of IBD, focusing on their diagnostic accuracy, methodological quality, and potential implications for clinical practice and future research.

## Review

Methodology

Study Design

This systematic review was conducted in accordance with the Preferred Reporting Items for Systematic Reviews and Meta-Analyses (PRISMA) guidelines [7] to ensure methodological rigor, transparency, and reproducibility. The review process was designed a priori, outlining the objectives, eligibility criteria, search strategy, and methods for data extraction and synthesis. All stages of the review, including study selection, data extraction, and quality assessment, were performed following standardized procedures to minimize bias.

The eligibility criteria for study inclusion were defined using the PICOS (Population, Intervention, Comparator, Outcomes, and Study Design) framework (Table 1). Studies were considered eligible if they evaluated the performance of deep learning models in the endoscopic assessment of disease severity in patients with IBD. Only studies published in the last five years (2021-2025) were included. This time frame was selected for two primary reasons. First, the objective of this review is to evaluate the current state-of-the-art performance of deep learning models in this domain, with direct relevance to contemporary clinical practice and near-future implementation. The period from 2021 onward has witnessed the rapid maturation of the field, including the widespread adoption of ViT architectures, self-supervised learning techniques, and large-scale multicenter validation studies, developments that have substantially improved model generalizability and clinical applicability. Second, while earlier pioneering studies provided foundational proof-of-concept evidence, many employed older CNN architectures (e.g., VGG-16, early ResNet variants) without external validation, and their performance metrics are not directly comparable to contemporary models trained on larger, more diverse datasets. To ensure that this review synthesizes evidence that reflects the current technical frontier and informs ongoing clinical implementation efforts, studies published before 2021 were excluded. However, readers should be aware that this decision prioritizes contemporary relevance over historical completeness, and key earlier studies are discussed in the Introduction and Discussion sections as contextual background.

**Table 1:** Study selection criteria as per the PICOS framework AUROC: area under the receiver operating characteristic curve

PICOS component	Description
Population (P)	Patients diagnosed with inflammatory bowel disease undergoing endoscopic evaluation
Intervention (I)	Deep learning models applied to endoscopic images or videos for severity assessment
Comparator (C)	Expert endoscopist assessment, histopathology, or validated endoscopic scoring systems
Outcomes (O)	Performance metrics including accuracy, sensitivity, specificity, AUROC, F1-score, or Dice coefficient
Study Design (S)	Original research studies including retrospective, prospective, and observational studies

Studies such as reviews, editorials, conference abstracts without full text, non-English publications, and studies not employing deep learning techniques were excluded.

Information Sources and Search Strategy

A comprehensive literature search was conducted across four major electronic databases: PubMed, Scopus, Embase, and Web of Science. The search strategy combined controlled vocabulary terms (e.g., MeSH and Emtree terms) and free-text keywords related to "inflammatory bowel disease", "endoscopy", "deep learning", "artificial intelligence", and "severity assessment". Boolean operators (AND, OR) were used to refine the search. Additionally, the reference lists of included studies were manually screened to identify any potentially relevant articles not captured in the initial search. The exact search strings used for each database are provided in the Appendices section.

Study Selection

All identified records were imported into EndNote X21 (Clarivate, London, UK) for organization and duplicate removal. After removing duplicates, titles and abstracts were independently screened by two reviewers to assess eligibility. Full-text articles of potentially relevant studies were then retrieved and evaluated against the predefined inclusion and exclusion criteria. Any disagreements between reviewers were resolved through discussion or consultation with a third reviewer to ensure consistency and accuracy in study selection.

Data Extraction

A standardized data extraction form was developed to systematically collect relevant information from each included study. Extracted data included study characteristics (author, year, country, study design), patient demographics, type of IBD, endoscopic modality, severity scoring system used, deep learning model architecture, dataset characteristics, validation methods, and reported performance metrics. Data extraction was performed independently by two reviewers to minimize errors and discrepancies. Any disagreements between reviewers were resolved through discussion or, if necessary, consultation with a third reviewer. Performance metrics (e.g., accuracy, area under the receiver operating characteristic curve (AUROC), F1-score) were extracted exactly as defined and reported in each primary study, without recalculation or standardization, to preserve the original authors' methodological choices.

Risk of Bias Assessment

The methodological quality and risk of bias of the included studies were assessed using the Quality Assessment of Diagnostic Accuracy Studies (QUADAS-2) tool [8]. This tool evaluates studies across four key domains: patient selection, index test, reference standard, and flow and timing. Each domain was assessed for risk of bias and applicability concerns. The assessment was conducted independently by two reviewers, and any disagreements were resolved through consensus. The results of the risk of bias assessment were summarized in a table, with each study rated as low, high, or unclear risk of bias across the four domains, accompanied by a narrative synthesis in the Results section.

Data Synthesis

A qualitative synthesis of the included studies was performed to summarize the performance and characteristics of deep learning models in endoscopic severity assessment of IBD. Key findings were organized by disease type (UC vs. CD), model architecture, and performance metrics (e.g., accuracy, AUROC, F1-score) to facilitate comparison across studies. A meta-analysis was not conducted due to substantial heterogeneity across studies. Specifically, the following types of heterogeneity precluded statistical pooling: (1) variation in reference standards (some studies used histopathology as ground truth, others relied on single-expert endoscopic scoring, and others employed multi-expert consensus annotations, each with different levels of inter-rater reliability); (2) variation in outcome definitions (studies reported different metrics (accuracy, AUROC, F1-score, Dice coefficient) and used different severity thresholds (e.g., binary mild vs. moderate-severe vs. four-grade MES classification)); (3) variation in data sources (models were trained on different endoscopic modalities (standard colonoscopy, video endoscopy, capsule endoscopy) with vastly different image resolutions and acquisition protocols); and (4) variation in validation methods (some used k-fold cross-validation, others used independent test sets, and only a subset performed external validation on separate datasets). Given this heterogeneity, conducting a meta-analysis could produce misleading pooled estimates and compromise the validity of conclusions; therefore, a narrative synthesis was deemed more appropriate to accurately interpret and contextualize the findings.

Results

Study Selection Process

**Figure 1:**
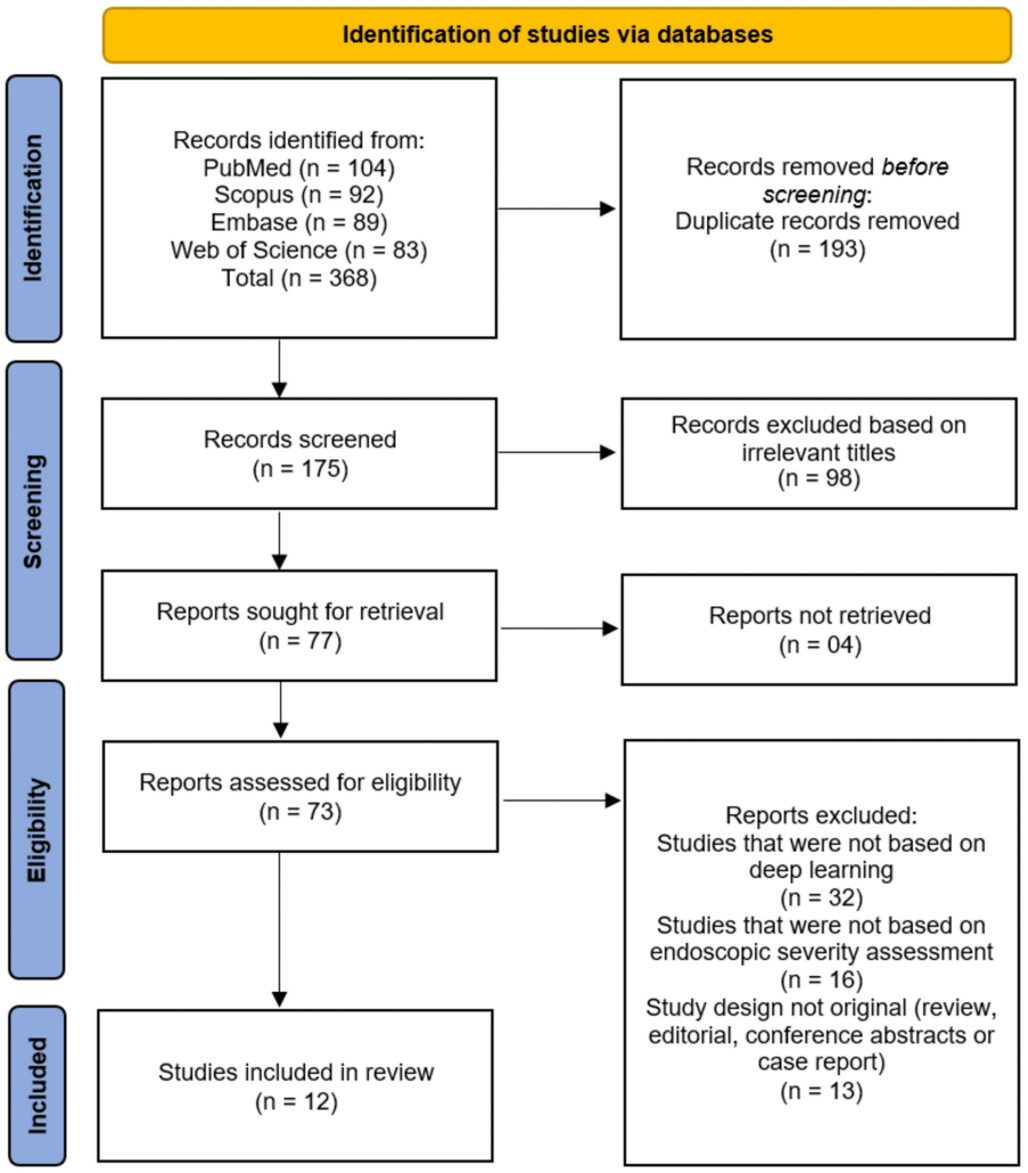
PRISMA flow diagram of the study selection process PRISMA: Preferred Reporting Items for Systematic Reviews and Meta-Analyses

A total of 368 records were initially identified through database searches, comprising 104 from PubMed, 92 from Scopus, 89 from Embase, and 83 from Web of Science. After the removal of 193 duplicate records, 175 records remained for title screening. Following the screening of titles, 98 records were excluded due to irrelevant titles, leaving 77 reports sought for retrieval. Of these, four reports could not be retrieved, resulting in 73 reports assessed for full-text eligibility. During the full-text eligibility assessment, 61 reports were excluded for the following reasons: 32 studies were not based on deep learning, 16 studies did not focus on endoscopic severity assessment, and 13 studies were not original research designs (including reviews, editorials, conference abstracts, or case reports). Consequently, a total of 12 studies [9-20] met the predefined inclusion criteria and were included in this systematic review (Figure 1).

Study Characteristics

A total of 12 studies [9-20], published between 2021 and 2025, met the inclusion criteria for this systematic review. The characteristics of these studies are summarized in Table 2. The included studies were geographically diverse, with contributions from the USA, Europe, and Asia, encompassing multicenter and single-center designs. The sample sizes varied considerably, ranging from 22 patients with over 700,000 patch images [14] to 2,411 patients in a large multicenter trial [9]. The studies focused on both UC and CD, with several including both [9,19]. Endoscopic modalities were primarily standard colonoscopy [10-13,20] and capsule endoscopy [14-19]. For severity scoring, the MES and Ulcerative Colitis Endoscopic Index of Severity (UCEIS) were used for UC [9-11,13,14,20], while the SES-CD was employed for CD [12]. A variety of deep learning architectures were utilized, ranging from CNNs like ResNet [13,14,18] and Visual Geometry Group (VGG) [13,17,20] to more advanced models such as ViT [9] and recurrent attention networks [17]. Validation methods were generally robust, with the majority employing k-fold cross-validation, independent test sets, and, in some cases, external validation on separate trials or datasets [9,12,20].

**Table 2:** Characteristics of the included studies AI: artificial intelligence; RCT: randomized controlled trial; UC: ulcerative colitis; IBD: inflammatory bowel disease; CD: Crohn's disease; HD-WLE: high-definition white light endoscopy; NBI: narrow band imaging; CCE-2: second-generation colon capsule endoscopy; SB: small bowel; MES: Mayo Endoscopic Score; UCEIS: Ulcerative Colitis Endoscopic Index of Severity; AI-CDS: AI-clinical decision support; PICaSSO: Paddington International Virtual Chromoendoscopy Score; SES-CD: Simple Endoscopic Score for Crohn's Disease; ViT: Vision Transformers; cycleGAN: Cycle-Consistent Adversarial Network; NN: neural network; GRU: gated recurrent unit; DB: database; CV: cross-validation; AUC: area under the curve; LOO: leave-one-out; GI: gastrointestinal; Se/Sp: sensitivity/specificity

Author (year)	Country	Study design	Sample size (patients/images)	IBD type	Endoscopic modality	Severity scoring system	Deep learning model	Data source	Validation method
Chaitanya et al., 2024 [9]	Multi-country	Multicenter (retro + prospective)	2411 pts; 4911 vids; 71M frames	UC + CD (UC for scoring)	Endoscopy videos	MES, UCEIS	ViT (ArgesFM) + Transformer (ArgesMES/UCEIS)	4 trials + QUASAR	4-fold CV + test + prospective
Stidham et al., 2026 [10]	USA	Post hoc RCT	387 patients	UC	Video endoscopy	MES, AI-CDS	AI model	TrueNorth trial	AUC, κ, Youden
Iacucci et al., 2025 [11]	UK + 11 intl centers	Prospective	iScan: 302/2535; NBI: 54/765	UC	HD-WLE, iScan2/3, NBI	UCEIS, PICaSSO	AI-switching (NN + cycleGAN)	iScan and NBI cohorts	Random test/val sets, 5-fold CV, expert review
Cai et al., 2026 [12]	USA	Phase 3b RCT	STARDUST: 307/4487; SEAVUE: 367	CD	Colonoscopy	SES-CD/CDS	U-Net	STARDUST/SEAVUE	3-fold CV + external
Sutton et al., 2022 [13]	Norway	Retrospective image-based	851 UC images (HyperKvasir); 8000 images (Kvasir v2)	UC	Standard colonoscopy	MES	InceptionV3, ResNet50, VGG19, DenseNet121	Kvasir and HyperKvasir datasets (Bærum Hospital)	5-fold CV + 20% test set
Higuchi et al., 2022 [14]	Japan	Single-center, retrospective	22 patients/739,021 patch images	UC	Capsule endoscopy (CCE-2)	MES (modified MES)	ResNet50 (CNN)	Hirosaki University Hospital	Independent validation dataset of 8 patients/255,377 images
Barash et al., 2021 [15]	Israel	Retrospective	17,640 images (1,242 train/248 test)	CD	Capsule endoscopy	3-grade (1-3)	Ordinal CNN (ResNet-based)	Single-center DB	5-fold CV + test set
Klang et al., 2021 [16]	Israel	Retrospective	27,892 images	CD	Capsule (SBIII)	Mild/Mod/Sev ulcers + strictures	EfficientNet-B5	Internal bank	10-fold CV + LOO
de Maissin et al., 2021 [17]	France	Retrospective multicenter	63/3498	CD	SB capsule endoscopy (PillCam SB3)	Lesion-based severity hierarchy	Recurrent attention CNN (VGG16 + GRU)	CrohnIPI dataset (3 centers)	70/10/20 split + 5-fold CV
Majtner et al., 2021 [18]	Denmark	Prospective multicenter trial	38/7744	CD	Capsule endoscopy (PillCam CE)	13-class lesion scale (normal-severe ulcers)	ResNet50 (transfer learning, ensemble)	3 Danish centers (NCT trials)	Random split (70/10/20) + patient-level split; independent test set; kappa + accuracy
Brodersen et al., 2024 [19]	Denmark	Prospective diagnostic accuracy (NCT03134586)	131/2.97M	CD, UC, IBD	Capsule endoscopy (PillCam Colon-2/Crohn)	Eliakim score	AXARO® DL system	3 Danish centers + CAD-CAP DB	Blinded GI expert reference; AUC, Se/Sp, kappa
Kim et al., 2023 [20]	South Korea	Retrospective cohort	492/984	UC	Colonoscopy	MES (0-3)	VGG16 CNN + auxiliary heads	Single center + HyperKvasir	12-fold CV, internal test, external test, clinician comparison

Performance of Deep Learning Models for UC

The performance of deep learning models for UC severity assessment is detailed in Table 3 [9-20]. Across multiple studies, models demonstrated a high capacity for accurate grading. In a large multicenter study, the ArgesFM model, a spatio-temporal transformer, achieved weighted F1-scores of up to 0.731 for UCEIS vascular pattern scoring on prospective validation, outperforming previous state-of-the-art CNN and self-supervised learning models [9]. Similarly, a VGG16-based CNN with auxiliary heads developed by Kim et al. showed strong performance in distinguishing MES 0 from 1, achieving a test AUROC of 0.9661 and an F1-score of 91.7%, outperforming novice endoscopists and generalizing well to an external dataset [20].

**Table 3:** Performance of deep learning models in endoscopic severity assessment ViT: Vision Transformers; MIL: multiple instance learning; AI: artificial intelligence; CDS: clinical decision support; HD-WLE: high-definition white light endoscopy; NBI: narrow band imaging; CVE: Crohn's volume of endoscopy; NN: neural network; GRU: gated recurrent unit; MES: Mayo Endoscopic Score; UC: ulcerative colitis; CD: Crohn's disease; CE: capsule endoscopy; SB: small bowel; IBD: inflammatory bowel disease; PMS: premenstrual syndrome; QoL: quality of life; UCEIS: Ulcerative Colitis Endoscopic Index of Severity; PICaSSO: Paddington International Virtual Chromoendoscopy Score; RHI: Robarts Histological Index; NHI: Nancy Histological Index; PHRI: PICaSSO Histologic Remission Index; SES-CD: Simple Endoscopic Score for Crohn's Disease; CDAI: Crohn's Disease Activity Index; AUC: area under the curve; AUROC: area under the receiver operating characteristic curve; NR: not reported; SOTA: state-of-the-art; CNN/SSL: convolutional neural network/self-supervised learning; Grad-CAM: gradient-weighted class activation mapping; Corr: correlation; Acc: accuracy; Sens: sensitivity; Spec: specificity; Dx: diagnostic

Author (year)	Model architecture	Task	Outcome measure	Accuracy (%)	Sensitivity (%)	Specificity (%)	AUROC	F1-score/Dice	Key findings
Chaitanya et al., 2024 [9]	ArgesFM (ViT-Base, DINOv2) + Transformer + Attention MIL	MES and UCEIS severity scoring (video-based)	Weighted F1-score	NR	NR	NR	NR	0.644 (UNIFI), 0.609 (JAKUC), 0.620 (QUASAR); up to 0.731 (UCEIS vascular)	Outperformed SOTA CNN/SSL models; strong generalization with prospective validation
Stidham et al., 2026 [10]	Unspecified AI (CDS)	Severity scoring (UC)	PMS, QoL, κ	NR	NR	NR	0.85	NR	Better than MES for remission and QoL; higher agreement; improved treatment detection
Iacucci et al., 2025 [11]	AI-switching multimodal DL (HD-WLE + iScan2 + iScan3 + NBI)	Endoscopic and histologic severity assessment and ER prediction	UCEIS, PICaSSO, RHI, NHI, PHRI	81-90	60-85	81-92	0.89-0.92	72-79	Multimodal model outperformed unimodal models; high agreement with human experts
Cai et al., 2026 [12]	U-Net (CVE)	Ulcer detection and CD severity	Dice, Corr with SES-CD/CDAI	NR	NR	NR	NR	Any: 0.591; mild: 0.393; mod-severe: 0.424	Outperformed humans; strong SES-CD correlation; separated clinical remission best for mod-severe ulcers
Sutton et al., 2022 [13]	InceptionV3, ResNet50, VGG19, DenseNet121	Diagnosis and grading	Binary	High	NR	NR	0.66-0.999	NR	High accuracy for diagnosis; DenseNet121 best for grading; Grad-CAM highlights UC areas
Higuchi et al., 2022 [14]	ResNet50	UC severity (patches)	MES0-MES3	Train 99.2, Val 98.3	NR	NR	NR	NR	High accuracy; MES3 slightly lower; enabled topographic colon maps
Barash et al., 2021 [15]	Ordinal CNN (ResNet-based)	Ulcer severity classification (CE, CD)	Acc, Sens, Spec, F1, AUC	62.4/78.0/91.0	34.2/73.4/91.0	71.2/91.2/91.3	0.565/0.939/0.958	0.278/0.835/0.912	Best for 1 vs. 3; weak for 1 vs. 2
Klang et al., 2021 [16]	EfficientNet-B5	Multi + binary classification (ulcers, strictures)	Acc, AUC	79.4 (overall); 93.5 (strictures)	92	89	0.971 (main); 0.849 (sub)	NR	High performance in stricture detection; excellent AUC vs. normal/ulcers
de Maissin et al., 2021 [17]	Recurrent Attention NN (GRU + VGG16) + ResNet-34, VGG16, VGG19	Binary classification (pathological vs. non-pathological SB lesions in CD)	Precision/Sens/Spec/Acc	Up to 94.58	Up to 92.09	Up to 94.76	NR	NR	Best performance achieved with multi-expert consensus labeling
Majtner et al., 2021 [18]	Ensemble ResNet50	IBD lesion classification and severity grading	Acc, Sens, Spec, κ	98.4-98.6	95.7-96.2	99.8-100	NR	NR	High accuracy for Crohn's lesion detection; strong agreement with experts
Brodersen et al., 2024 [19]	AXARO® DL framework	CD/IBD detection from CE	Dx performance vs. expert	NR	CD: 92-96; IBD: 97	CD: 90-93; IBD: 90-91	0.91-0.94	NR	97% image reduction; 87-94% faster review; high agreement; strong CD/IBD detection performance
Kim et al., 2023 [20]	VGG16-based CNN with dual auxiliary + fusion classifier	MES classification (UC endoscopy, colon + rectum images)	Acc, AUROC, F1	NR	NR	NR	0.9661 (test), 0.8587 (external)	91.7% (test)	Best backbone; auxiliary heads improved performance; outperformed novices; strong generalization on external dataset

For video-based analysis, Stidham et al. demonstrated that an AI model could outperform conventional MES scoring by achieving higher agreement with patient-reported outcomes and quality-of-life measures in a phase 3 UC trial [10]. The AI model also showed a higher correlation with clinical remission, suggesting it may be a more clinically relevant endpoint. Higuchi et al. applied a ResNet50 model to colon capsule endoscopy images, achieving a high validation accuracy of 98.3% for classifying MES grades, which enabled the creation of topographic maps of disease severity [14].

A novel approach by Iacucci et al. involved an AI-switching model that generated simultaneous multimodal images (high-definition white light endoscopy (HD-WLE), iScan, narrow band imaging (NBI)). This model demonstrated high accuracy (81-90%), AUROC (0.89-0.92), and F1-scores (72-79%) for predicting both endoscopic and histologic severity, outperforming unimodal models and showing high agreement with human experts [11].

Performance of Deep Learning Models for CD

The application of deep learning for CD severity assessment also yielded promising results (Table 3). Cai et al. developed a U-Net model (Crohn's volume of endoscopy (CVE)) to quantify mucosal ulceration in CD. The model outperformed human readers in correlating with SES-CD and showed the best performance for distinguishing moderate-to-severe ulcers, with a Dice score of 0.424 [12]. In the context of capsule endoscopy, an ordinal CNN by Barash et al. performed best at distinguishing between mild and severe ulcers (AUC of 0.939 and 0.958 for 1 vs. 3, respectively) but was weaker at differentiating adjacent severity grades (e.g., 1 vs. 2) [15].

For lesion detection and classification, Klang et al. used an EfficientNet-B5 model to detect ulcers and strictures in CD, achieving a high overall accuracy of 93.5% for stricture detection and an excellent AUROC of 0.971 for the primary task [16]. Similarly, de Maissin et al. found that a recurrent attention neural network (VGG16 + gated recurrent unit (GRU)) achieved up to 94.6% accuracy for binary classification of pathological small bowel lesions when trained on a multi-expert consensus dataset [17]. An ensemble ResNet50 model by Majtner et al. also showed very high accuracy (98.4-98.6%) and strong agreement with experts for detecting and grading CD lesions in capsule endoscopy images [18].

Overall Diagnostic Accuracy and Clinical Utility

Beyond specific severity grading, several studies evaluated the broader diagnostic utility of AI models (Table 3). Sutton et al. compared four different CNN architectures, finding that DenseNet121 was most effective for grading UC, with all models achieving high accuracy for diagnosis [13]. The AXARO® DL system evaluated by Brodersen et al. demonstrated high diagnostic performance for CD (sensitivity 92-96%; specificity 90-93%) and IBD (sensitivity 97%) on capsule endoscopy. Critically, it enabled a 97% reduction in the number of images requiring expert review and resulted in 87-94% faster reading times, highlighting a significant clinical workflow benefit [19]. Overall, the findings across these 12 studies consistently show that deep learning models achieve high accuracy, sensitivity, and specificity for a range of tasks, from diagnosing IBD to grading its endoscopic severity, with many models demonstrating robust generalization through external validation.

Risk of Bias Assessment

The risk of bias for the 12 included studies was evaluated using the QUADAS-2 tool, with a detailed summary presented in Table 4. Overall, the majority of studies demonstrated a low risk of bias across the four key domains. Nine studies were judged to have an overall low risk of bias [9,11,12,14,16-20], reflecting rigorous methodology in patient selection, index test implementation, reference standard application, and flow and timing. Three studies had items rated as unclear. In the domain of patient selection, Sutton et al. [13] was rated as unclear because the study used publicly available datasets (HyperKvasir and Kvasir v2) without explicitly reporting the selection criteria for included images, making it impossible to determine whether case selection was consecutive or if preprocessing introduced bias. For the index test domain, Stidham et al. [10] was rated as unclear due to a lack of specification regarding the AI model architecture (e.g., network type, layers, or hyperparameters), preventing the assessment of whether the index test was conducted without knowledge of the reference standard. In the reference standard domain, Barash et al. [15] was rated as unclear because the reference standard was based on a single expert annotator without any reported inter-rater reliability or intraobserver agreement, raising uncertainty about the reproducibility of severity grading. All studies were rated as low risk for flow and timing, as they consistently applied the same reference standard to all participants and included all patients in the final analysis. Concerning applicability concerns, all 12 studies were rated as low, indicating that the patient populations, index tests, and reference standards were appropriately aligned with the review question (Table 4).

**Table 4:** Risk of bias assessment using QUADAS-2 QUADAS-2: Quality Assessment of Diagnostic Accuracy Studies

Author (year)	Domain 1: patient selection	Domain 2: index test	Domain 3: reference standard	Domain 4: flow and timing	Overall risk of bias	Applicability concerns
Chaitanya et al., 2024 [9]	Low	Low	Low	Low	Low	Low
Stidham et al., 2026 [10]	Low	Unclear	Low	Low	Unclear	Low
Iacucci et al., 2025 [11]	Low	Low	Low	Low	Low	Low
Cai et al., 2026 [12]	Low	Low	Low	Low	Low	Low
Sutton et al., 2022 [13]	Unclear	Low	Low	Low	Unclear	Low
Higuchi et al., 2022 [14]	Low	Low	Low	Low	Low	Low
Barash et al., 2021 [15]	Low	Low	Unclear	Low	Unclear	Low
Klang et al., 2021 [16]	Low	Low	Low	Low	Low	Low
de Maissin et al., 2021 [17]	Low	Low	Low	Low	Low	Low
Majtner et al., 2021 [18]	Low	Low	Low	Low	Low	Low
Brodersen et al., 2024 [19]	Low	Low	Low	Low	Low	Low
Kim et al., 2023 [20]	Low	Low	Low	Low	Low	Low

Discussion

Summary of Main Findings

This systematic review synthesized evidence from 12 studies [9-20] published between 2021 and 2025 to evaluate the performance of deep learning models in endoscopic severity assessment of IBD. The findings demonstrate that deep learning models, spanning a range of architectures from CNNs to ViT, consistently achieve high diagnostic accuracy, sensitivity, and specificity across both UC and CD. These models have shown particular promise in grading endoscopic severity, detecting mucosal ulcers, identifying strictures, and even predicting histologic outcomes, with several studies reporting performance that rivals or exceeds that of human experts. The robustness of these findings is supported by the use of rigorous validation methods, including k-fold cross-validation, independent test sets, and, in a subset of studies, external validation on separate clinical trial datasets. Collectively, the evidence suggests that deep learning has matured from a proof-of-concept technology to a clinically viable tool capable of enhancing endoscopic assessment in IBD.

Performance in UC

A key finding of this review is the high performance of deep learning models for UC severity grading using established endoscopic indices. The ArgesFM model, a spatio-temporal transformer, achieved weighted F1-scores of up to 0.731 for UCEIS vascular pattern scoring and outperformed previous state-of-the-art models on prospective validation, demonstrating the value of leveraging temporal information from video data rather than relying solely on static images [9]. This aligns with the broader trend in medical imaging where sequential information enhances diagnostic accuracy, a concept supported by prior work demonstrating that video-based analysis improves the detection of subtle mucosal changes compared to isolated frame assessment. Similarly, Kim et al. reported a VGG16-based CNN with auxiliary heads that achieved an AUROC of 0.9661 for distinguishing MES 0 from 1, outperforming novice endoscopists and generalizing well to an external dataset [20]. This is particularly clinically relevant because differentiating between normal mucosa and mild inflammation remains a common challenge in clinical practice, where interobserver variability among endoscopists is well documented. The ability of deep learning to make this distinction with high accuracy suggests that AI could serve as a valuable adjunct, particularly for less experienced practitioners.

Performance in CD

For CD, the findings are equally compelling, though the body of evidence is somewhat smaller. Cai et al. developed a U-Net model to quantify mucosal ulceration, demonstrating that AI could outperform human readers in correlating with the SES-CD, particularly for moderate-to-severe ulcers [12]. This is noteworthy because CD is often characterized by patchy and discontinuous inflammation, making standardized endoscopic assessment more challenging than in UC. The study by Klang et al. further extended the capabilities of deep learning by detecting both ulcers and strictures in capsule endoscopy images, achieving an AUROC of 0.971 for stricture detection [16]. Strictures represent a critical complication in CD associated with significant morbidity, and their early detection is paramount. The high accuracy achieved in this study suggests that AI could facilitate the earlier identification of strictures, potentially enabling more timely therapeutic intervention. Moreover, the work by de Maissin et al. highlights the importance of high-quality reference standards, as their recurrent attention network achieved optimal performance when trained on multi-expert consensus annotations rather than single-expert labels [17]. This underscores a fundamental principle in AI development: model performance is intrinsically limited by the quality of the ground truth data, and efforts to reduce interobserver variability through expert consensus are likely to yield more robust and generalizable models.

Clinical Utility and Workflow Efficiency

The clinical utility of these models extends beyond mere diagnostic accuracy. Brodersen et al. provided compelling evidence of workflow efficiency gains, demonstrating that the AXARO® DL system enabled a 97% reduction in the number of images requiring expert review and resulted in 87-94% faster reading times for capsule endoscopy [19]. This finding addresses one of the most significant barriers to the widespread adoption of capsule endoscopy: the time-intensive nature of image review. By substantially reducing the burden on clinicians, AI may unlock the full potential of capsule endoscopy as a first-line diagnostic tool for IBD. Similarly, Stidham et al. demonstrated that an AI model could outperform conventional MES scoring by achieving higher agreement with patient-reported outcomes and quality-of-life measures [10]. This finding is particularly important because it suggests that AI-based assessments may be more clinically meaningful than traditional endoscopic scores, which were not originally designed to capture the patient experience. The alignment of AI-derived metrics with patient-centered outcomes represents a paradigm shift that could reframe how endoscopic remission is defined and measured in clinical trials and practice.

Real-Time Clinical Application: Current Status and Barriers

A critical question for translating these findings into practice is whether any of the included studies evaluated deep learning models in real-time during live endoscopy. After careful review of all 12 studies, none implemented true real-time inference during ongoing procedures. All studies were retrospective analyses of previously recorded still images or video recordings [9-20]. For example, Chaitanya et al. applied their transformer model to endoscopy videos from completed trials [9]; Sutton et al. analyzed static images from public datasets [13]; and Higuchi et al. evaluated colon capsule endoscopy images after the completion of capsule passage [14]. Even studies using video data processed the recordings offline rather than providing live feedback to endoscopists during procedures.

This distinction is important because real-time implementation presents several practical challenges not addressed by retrospective studies. First is computational requirements: real-time analysis demands low-latency processing (typically <100-200 milliseconds per frame) and sufficient on-site computing power, which may require dedicated hardware (e.g., graphics processing unit (GPU)-equipped workstations) not yet standard in many endoscopy suites. Second is integration with existing endoscopic equipment: proprietary video output formats and varying image resolutions across different endoscope manufacturers create technical compatibility issues that require customized solutions. Third is regulatory and legal barriers: real-time AI systems used for clinical decision-making require regulatory approval (e.g., FDA Class II or III clearance, Conformité Européenne (CE, European Conformity) marking under Medical Device Regulation (MDR)) and raise questions about legal liability if the AI misses a finding or provides an incorrect severity grade. None of the included studies addressed these implementation barriers. Therefore, while the diagnostic accuracy reported is highly encouraging, immediate clinical application remains in the near future rather than the current reality. Prospective studies with real-time inference workflows and integration into endoscopic towers are urgently needed before widespread adoption can be recommended.

Comparison With Existing Literature

When comparing these findings to the broader literature, several consistent themes emerge. A review by Stidham and Higgins published in 2018 highlighted the limitations of conventional endoscopic scoring systems, including high interobserver variability and weak correlation with symptoms [21]. Our findings extend this work by demonstrating that deep learning models can overcome many of these limitations, offering more consistent and potentially more clinically relevant assessments. More recently, a review by Sundaram et al. emphasized the potential of AI to standardize endoscopic assessment in IBD clinical trials, noting that the integration of AI could reduce sample size requirements and improve trial efficiency [22]. Our findings support this assertion, particularly given the strong performance of models like ArgesFM across multiple trial datasets [9]. Additionally, a study by Rimondi et al. demonstrated that AI-based assessment of endoscopic severity correlated more strongly with histologic healing than traditional endoscopic scores [23], a finding echoed in our review by Iacucci et al., whose multimodal model predicted both endoscopic and histologic severity with high accuracy [11].

Diversity of Deep Learning Architectures

The diversity of deep learning architectures represented in this review reflects the rapid evolution of the field. Early studies predominantly employed CNN-based architectures such as ResNet, VGG, and DenseNet [13,14,18], which have become the workhorses of medical image analysis. More recent studies have adopted advanced architectures including ViT [9], recurrent attention networks [17], and ensemble methods [18], each offering distinct advantages. ViT, in particular, have gained traction for their ability to capture global contextual relationships that may be missed by CNNs, which are inherently limited by their local receptive fields. The superior performance of ArgesFM, which combines a ViT with spatio-temporal attention, suggests that future developments will likely continue to leverage hybrid architectures that integrate multiple complementary approaches. Notably, the successful application of these models across different endoscopic modalities, including standard colonoscopy, video endoscopy, and capsule endoscopy, demonstrates the flexibility and generalizability of deep learning approaches in this domain.

Considerations for Clinical Integration

Despite these promising findings, several important considerations must be acknowledged. First, the heterogeneity in model architectures, validation methods, and outcome measures across studies limits the ability to draw direct comparisons. While most studies reported accuracy, sensitivity, and specificity, the definitions of these metrics varied, and several studies did not report key performance indicators such as AUROC or F1-score consistently. This variability highlights the need for standardized reporting guidelines specific to AI in medical imaging, such as the Transparent Reporting of a multivariable prediction model for Individual Prognosis Or Diagnosis + artificial intelligence (TRIPOD-AI) and Standards for Reporting of Diagnostic Accuracy Studies using Artificial Intelligence (STARD-AI) statements, which aim to improve transparency and reproducibility. Second, while external validation was performed in a subset of studies [9,12,20], many models have not been tested on diverse, multicenter datasets from different geographic regions and healthcare settings. The generalizability of these models to populations and endoscopy systems not represented in training data remains an open question. Moreover, deep learning model performance can be sensitive to variations in endoscopes and imaging systems across different hospitals and manufacturers, and acknowledging this variability is essential when considering real-world deployment. Third, the clinical integration of these models has not been extensively studied. Only one study explicitly reported on workflow efficiency gains [19], and none evaluated the impact of AI-assisted reading on clinical decision-making or patient outcomes in real-world settings. Practical challenges to clinical implementation include data privacy and security regulations (e.g., General Data Protection Regulation (GDPR), Health Insurance Portability and Accountability Act (HIPAA)), the need for regulatory approval processes (e.g., FDA, CE marking), and the requirement for standardized training programs to ensure endoscopists can use these AI tools effectively. Implementation science research will be essential to understand how these tools can be effectively integrated into clinical practice without disrupting existing workflows.

Reference Standard Considerations

Another important consideration relates to the reference standard used in these studies. While most employed established endoscopic scoring systems are interpreted by experienced gastroenterologists, these standards themselves are subject to variability. The findings from de Maissin et al., where model performance improved with multi-expert consensus annotations, underscore the importance of addressing this issue [17]. Future studies should consider using consensus-based or central reading approaches to minimize reference standard bias. Additionally, the relationship between AI-derived scores and clinically meaningful endpoints such as long-term outcomes, need for surgery, or hospitalization has not been adequately explored. Longitudinal studies linking AI-based assessments to patient outcomes will be critical to establish the clinical value of these tools beyond their immediate diagnostic accuracy.

Limitations

This systematic review has several limitations that should be acknowledged. First, the search was limited to studies published in English, which may have introduced language bias and excluded relevant research published in other languages. Second, the heterogeneity across studies in terms of model architectures, validation approaches, and outcome measures precluded a meta-analysis, limiting the ability to derive pooled estimates of diagnostic performance. Additionally, variability in how performance metrics such as accuracy and F1-score were calculated and reported across studies (e.g., weighted vs. macro F1-score, binary vs. multi-class accuracy) further contributes to the overall heterogeneity and limits direct comparability of findings. Third, publication bias is a potential concern, as studies reporting positive results are more likely to be published than those with negative or null findings. Fourth, the quality assessment using QUADAS-2 revealed that three studies had unclear risk of bias due to insufficient reporting of patient selection, index test details, or reference standard methodology, which may affect the overall confidence in these findings. Fifth, the review focused exclusively on endoscopic severity assessment and did not include studies evaluating histologic or radiographic AI applications, which represent important complementary approaches to IBD assessment. Finally, the rapid pace of innovation in deep learning means that some of the included studies, particularly those published in 2021, may already be superseded by more recent advances not captured in this review.

## Conclusions

Deep learning models consistently achieve high performance in endoscopic severity assessment of IBD, with several models rivaling or exceeding human expert performance. The evidence supports the potential of AI to standardize endoscopic scoring, improve correlation with patient-centered outcomes, and enhance clinical workflow efficiency. Transformer-based architectures and models leveraging spatio-temporal information from video data represent promising directions for future development. However, important gaps remain, including the need for rigorous external validation across diverse populations, standardized reporting of model performance, and prospective studies evaluating clinical implementation and impact on patient outcomes. As deep learning technologies continue to evolve, their integration into routine clinical practice for IBD assessment appears increasingly feasible, with the potential to transform both clinical care and research paradigms. Future work should prioritize the development of generalizable models, the establishment of standardized evaluation frameworks, and the generation of evidence linking AI-based assessments to long-term patient outcomes.

## Appendices

**Table 5:** Exact search strings used for each database

Database	Search string
PubMed	(("inflammatory bowel disease"[MeSH Terms] OR "inflammatory bowel disease"[Title/Abstract] OR "ulcerative colitis"[Title/Abstract] OR "Crohn disease"[MeSH Terms] OR "Crohn's disease"[Title/Abstract]) AND ("endoscopy"[MeSH Terms] OR "endoscopy"[Title/Abstract] OR "capsule endoscopy"[Title/Abstract] OR "colonoscopy"[Title/Abstract]) AND ("deep learning"[MeSH Terms] OR "deep learning"[Title/Abstract] OR "artificial intelligence"[MeSH Terms] OR "artificial intelligence"[Title/Abstract] OR "neural networks, computer"[MeSH Terms] OR "convolutional neural network"[Title/Abstract] OR "machine learning"[Title/Abstract]) AND ("severity"[Title/Abstract] OR "severity assessment"[Title/Abstract] OR "disease severity"[Title/Abstract] OR "grading"[Title/Abstract])) AND (2021/01/01:2025/12/31[Date - Publication])
Scopus	( TITLE-ABS-KEY("inflammatory bowel disease" OR "ulcerative colitis" OR "Crohn's disease") AND TITLE-ABS-KEY("endoscopy" OR "capsule endoscopy" OR "colonoscopy") AND TITLE-ABS-KEY("deep learning" OR "artificial intelligence" OR "convolutional neural network" OR "machine learning" OR "neural network") AND TITLE-ABS-KEY("severity" OR "severity assessment" OR "disease severity" OR "grading") ) AND PUBYEAR > 2020 AND PUBYEAR < 2026
Embase	('inflammatory bowel disease'/exp OR 'inflammatory bowel disease':ab,ti OR 'ulcerative colitis':ab,ti OR 'crohn disease'/exp OR 'crohn's disease':ab,ti) AND ('endoscopy'/exp OR 'endoscopy':ab,ti OR 'capsule endoscopy':ab,ti OR 'colonoscopy':ab,ti) AND ('deep learning'/exp OR 'deep learning':ab,ti OR 'artificial intelligence'/exp OR 'artificial intelligence':ab,ti OR 'convolutional neural network':ab,ti OR 'machine learning':ab,ti) AND ('severity':ab,ti OR 'severity assessment':ab,ti OR 'disease severity':ab,ti OR 'grading':ab,ti) AND (2021-2025)/py
Web of Science	((TS=("inflammatory bowel disease" OR "ulcerative colitis" OR "Crohn's disease")) AND (TS=("endoscopy" OR "capsule endoscopy" OR "colonoscopy")) AND (TS=("deep learning" OR "artificial intelligence" OR "convolutional neural network" OR "machine learning" OR "neural network")) AND (TS=("severity" OR "severity assessment" OR "disease severity" OR "grading"))) AND PY=(2021-2025)
